# Engagement barriers and service inequities in the NHS Breast Screening Programme: Views from British-Pakistani women

**DOI:** 10.1177/0969141319887405

**Published:** 2019-12-02

**Authors:** Victoria G Woof, Helen Ruane, Fiona Ulph, David P French, Nadeem Qureshi, Nasaim Khan, D Gareth Evans, Louise S Donnelly

**Affiliations:** 1 Division of Psychology & Mental Health, Faculty of Biology, Medicine and Health, School of Health Sciences, University of Manchester, Manchester, UK; 2 Nightingale & Prevent Breast Cancer Research Unit, Manchester University NHS Foundation Trust (MFT), Manchester, UK; 3 NIHR School of Primary Care, School of Medicine, University Park, Nottingham, UK; 4 Department of Genomic Medicine, Division of Evolution and Genomic Science, MAHSC, University of Manchester, Manchester University NHS Foundation Trust, Manchester, UK

**Keywords:** Breast screening, underserved populations, barriers, inequity, experiences

## Abstract

**Objectives:**

Previous research has largely attempted to explore breast screening experiences of South Asian women by combining opinions from Pakistani, Bangladeshi, and Indian women. This research often fails to reach the most underserved sub-groups of this population, with socioeconomic status not routinely reported, and English fluency being a participation requirement. With uptake low amongst British-Pakistani women, this study explores the experiences these women encounter when accessing the NHS Breast Screening Programme.

**Methods:**

19 one-to-one semi-structured interviews were carried out with British-Pakistani women from East Lancashire, UK. 14 interviews were conducted via an interpreter.

**Results:**

Data were analysed using thematic analysis. Three themes were identified: *‘Absence of autonomy in screening and healthcare access’* describes how currently the screening service does not facilitate confidentiality or independence. Access requires third-party intervention, with language barriers preventing self-expression. *‘Appraisal of information sources’* makes distinctions between community and NHS communication. Whereas community communication was invaluable, NHS materials were deemed inaccessible due to translation incongruences and incomprehensible terminology. *‘Personal suppositions of breast screening’* explores the subjective issues associated with disengagement, including, the cultural misalignment of the service, and perceiving screening as a symptomatic service.

**Conclusions:**

British-Pakistani women face some unique challenges when accessing breast screening. To promote uptake, the service needs to address the translation of screening materials and optimize upon community networks to disseminate knowledge, including knowledge of the screening environment within the context of culture to promote informed choice about attendance.

## Introduction

The number of South Asian women diagnosed with breast cancer in the UK is comparatively lower than figures recorded for the white population.^1^ Breast cancers that are detected in South Asian women are often diagnosed at a more progressive stage, resulting in a poorer prognosis and invasive treatments.^2–5^ To identify early stage cancers, women aged 50–70 in the UK are invited for three-yearly mammograms, through the National Health Service Breast Screening Programme (NHSBSP). However, over a 10-year period, uptake to the NHSBSP has fallen.^6^ With breast cancer risk factors (i.e. being overweight or obese) increasing in the current screening age population, this persistent decline in uptake is concerning.^7^

South Asian women in particular have lower screening attendance rates than host country women, and other minority ethnic communities.^7–12^ Insufficient knowledge and a lack of awareness about breast cancer and screening have been identified as significant barriers to uptake in South Asian communities.^5,13–16^ Emotional barriers and the anxiety of a diagnosis are also major deterrents.^5^,^13^,^16^,^17^ With modesty and respect highly valued amongst South Asian communities, screening and the discussion of breast health is often viewed as dishonourable, contributing to screening disengagement and a lack of family and community dialogue about breast cancer.^5^,^15^,^16^,^18^

To aid accessibility and informed choice, NHS screening programmes provide information in various languages. Despite this, verbatim translations can produce inaccuracies, causing a reduction in the amount of information disseminated, and lacking the desired impact apparent in the original English.^18^ With regards to verbal communication, healthcare professionals surveyed in 95 UK breast screening sites rated their ability to communicate verbally with South Asian woman as difficult and poor.^19^ These findings are consistent with data from UK Black and Minority Ethnic cancer patients (including South Asian women), who described themselves as unable to effectively access healthcare services due to a lack of confidence in healthcare professionals, as well as significant knowledge and communication barriers.^20^

The ethnicity of women attending breast screening is not routinely recorded by the NHS, resulting in a significant gap in knowledge regarding the disparities in screening uptake amongst minority ethnic communities.^21–23^ Survey data suggest that uptake is particularly poor amongst British-Pakistani women.^10^,^24^ The British-Pakistani population are the second largest minority ethnic group in England (approximately 1,112,282 residents), and the largest minority group in North West England.^25^ Compared with other South Asian populations settled in England (i.e. Bangladeshi and Indian), 30.9% of British-Pakistanis live in the 10% most deprived neighbourhoods, 19% of whom live in health deprived areas.^26^ According to the 2011 census, 185,925 British-Pakistanis in England have fair to very bad health. The proportion of British-Pakistanis described as having very bad health is higher than that recorded for other South Asian communities in England.^27^ Therefore, compared with other UK South Asian populations, British-Pakistani communities are of particular importance to health research, due to their level of health deprivation and poorer health outcomes.

Most research exploring the links between ethnicity and breast screening engagement has typically treated South Asian women as a homogenous group, generalizing findings from women of Pakistani, Bangladeshi, and Indian origins. Moreover, a scoping review of the literature has also revealed that the socioeconomic backgrounds of these women are *either* not reported or the most underserved populations are not reached.^13^,^14^,^17^,^18^ Furthermore, with some studies requiring fluency in English,^14^,^17^,^18^ an exploration into the screening experiences encountered by the most vulnerable and underserved sub-groups of this population is lacking. With changes being proposed to the programme regarding the introduction of breast cancer risk estimation,^28^ it is increasingly important to address and explore, *in-depth*, the current engagement barriers these women encounter before substantial service level changes are implemented. The aim of this study was specifically to explore the views and experiences that British-Pakistani women have of the NHSBSP.

## Methods

### Study design

This study adopted a qualitative cross-sectional design. Semi-structured interviews, conducted in English or via an interpreter, explored women’s views and experiences of the NHSBSP. Semi-structured interviews were chosen to obtain in-depth and nuanced insights from an underserved population, who have previously received little research attention in this area. Open-ended questions were used flexibly to enable participants to answer freely and to explore areas of personal meaning and interest.

### Recruitment

Participants were eligible to take part if they were British-Pakistani, and from a low socioeconomic background. Demographic details (i.e. ethnicity and postcode) were collected to ensure that women were from the identified population. Women were excluded if they were not born biologically female. Women with breast cancer or previous breast cancers were also excluded. Women approaching screening age were eligible to take part, although the researchers aimed to interview women of screening age. The rationale for including women approaching screening age was to gain their views on breast screening, their intentions to attend, and the challenges they could face prior to their attendance. Additionally, in some areas of England, an age-extension trial is taking place, so some women are invited for breast screening at age 47. Finally, women were not excluded based on their English language skills, as interpreters were to be employed if women requested their assistance.

To devise an optimal recruitment strategy for engaging women who are often described as ‘hard to reach’, Patient and Public Involvement meetings were set up prior to the study commencing. In these meetings, the study team were advised that direct contact via community networks would help to raise awareness of the research. Traditional methods of sending study information to women’s homes would prove ineffective, as many would be unlikely to access the information due to language barriers. Liaising with women via community networks was described as an effective way to establish trust. Additional advice was also provided on how to present the study to women. For example, it was suggested that describing the study as ‘research’, and wearing official NHS uniforms or University/NHS lanyards could be intimidating.

With this advice, participants were recruited from the East Lancashire area via a breast cancer community event, and community contacts. A member of the research team (a Genetic Counsellor, specializing in minority ethnic groups) provided contact details for outreach workers in the East Lancashire area to facilitate recruitment. The breast cancer community event was designed as a get together for women in the local area to learn more about breast screening. Culturally appropriate information materials were made available, and women were able to ask questions about breast cancer and screening. Casual discussions about the study were conducted, and information about participation was distributed. NHS-approved interpreters who were employed across the East Lancashire area were also present. Contact information was exchanged with the interpreters for future bookings for the study. As well as the community event, community contacts disseminated information about the study to women in the area. The research team were provided with the contact details and language preferences of women interested in taking part. When arranging a suitable time and date to be interviewed, a telephone interpreter was arranged for women who were not fluent in English.

The East Lancashire area was the chosen recruitment site as there is a large British-Pakistani community,^25^ a high proportion of neighbourhoods in the 10% most deprived in the UK,^29^ and uptake to breast screening is below the targeted 70%.^30^

### Participants

Nineteen women born in Pakistan who moved to the UK with their husbands and families were interviewed. All were living in high deprivation areas according to the Indices of Multiple Deprivation Index. Twelve women were of breast screening age (50+), five were aged under 50, and two did not disclose their ages. All women known to be of screening age reported previously attending for breast screening.

### Data collection

In one-to-one, semi-structured interviews lasting 1–2 hours, women were asked about their perceptions, knowledge, and experiences (if appropriate) of breast screening. For 14 women, an interpreter was employed. For the remaining women, an interpreter was declined, due to their fluency in English. The interviewer spoke directly to the interviewee, and the interpreter translated in third person. On one occasion the interviewee’s daughter interpreted for her mother, in addition to the employed interpreter. At the interviewee’s request, one interview was not recorded, and notes were taken instead. Data from this interview were synthesized into the larger dataset, to assess parity across accounts. The remaining interviews were recorded and transcribed verbatim. Personal details were anonymized, and participants were allocated pseudonyms.

### Analysis

Analysis was conducted in NVivo Version 11 using thematic analysis. For this thematic analysis a realist approach was adopted, with the researchers accepting that participants' accounts represented their experiences and realities.^31^ Primary data analysis was conducted by VGW with collaborative input from LSD, HR, and FU. Analysis began through familiarization, with researchers (VGW and LSD) reading each transcript in turn, after which coding began (conducted by VGW). To avoid the influence of pre-existing theory and literature, a manifest-inductive approach was adopted. As a realist epistemology was applied to this thematic analysis, data analysis was driven by the assumption that there is an apparent reality of the participant’s experiences inherent in the dataset.^31^ Coding was iterative; as new data were analysed, codes became more refined and initial themes were established. Data sufficiency for addressing the research aims was achieved, and recruitment stopped, when there appeared to be no new content being discussed in the final two interviews. Codes and themes were continually refined and assessed by VGW, LSD, HR, and FU to establish their representativeness across the dataset.

### Ethical approval and consent

This project was approved by London-City and East NHS Research Ethics Committee (ref: 17/LO/1275) and received HRA approval. The study was conducted in accordance with Good Clinical Practice guidelines and the Declaration of Helsinki. The study and consent form was explained fully by the interpreter and all participants gave written informed consent. Written informed consent was obtained from participants to use anonymized extracts from interviews in publications and presentations.

## Results

Data were grouped into three themes: (i) absence of autonomy in screening and healthcare access, (ii) appraisal of information sources, and (iii) personal suppositions of breast screening.

### Theme 1: Absence of autonomy in screening and healthcare access

#### Subtheme 1a: Preventing confidentiality

It was discussed by the women that a large majority of British-Pakistani women of breast screening age have limited/no literacy in English. Thus inviting women to breast screening via letter can be problematic. Some explained that they rely on family members, or women in their community, to explain the letter. For some, asking others to translate caused them to feel burdensome, and also concerned as to what effect translating private letters (which may contain results) could have on their translators, especially when the majority of those translating for them are their daughters, ‘*Mum does prefer in Urdu ‘cause she wouldn’t like to basically worry the kids ‘cause it affects their studies and stuff like that, so she’d rather keep that information to herself’* (*Fareeda via interpreter*). Some women acknowledged that relying on others to translate resulted in feelings of shame, due to their lack of English skills. To mitigate this, women suggested providing invitation/result letters in languages in which they are literate, to afford them privacy and independence: *‘But, if they could do it in a language, they don’t have to rely on anybody […] it’s nice to be independent, rather than saying, you know what, I’ve got this poor quality inside me, which there’s nothing I can do about’* (*Sadia via interpreter*). It was acknowledged, however, that some members of the British-Pakistani community, especially older generations, cannot read or write, and so providing information in their spoken language would not necessarily mitigate this barrier to privacy.

#### Subtheme 1b: Insufficient opportunity for self-expression

Some women explained that, during medical appointments, including breast screening, they are often unable to communicate with their healthcare professional due to limited fluency in English. In one account, a woman who had moved to the UK from Pakistan recalled the struggles she encountered when attending GP appointments, ‘*Six years she really struggled. She couldn’t explain anything to her doctor, and just felt really bad […] there was no way that she could explain to him what was wrong with her, and he couldn’t understand her either. There weren’t any interpreters, that she was aware of, that could help her*’ (*Roshanah via interpreter*). To compensate for this, women attend appointments with a family member who is able to translate and ‘speak for them’. Using family members as translators has the drawback of putting them in an uncomfortable position, resulting in them being selective in what they are willing to ask on the woman’s behalf, *‘Yeah, she thinks that we get embarrassed […] Like we go to the doctor with her and she’ll say, ask him this, and sometimes we won’t. We’ll be like, no, I don’t want to ask that’ *(*Fareeda via her daughter acting as interpreter*). Unaware that interpretation services exist, women expressed that a professional interpreter should be employed across all NHS services, so that women could be afforded the opportunity to express themselves without restrictions.

### Theme 2: Appraisal of information sources

#### Subtheme 2a: Invaluable social communication

It was highlighted that for this group of women, family and community dialogue is invaluable for sharing knowledge about breast screening. Women emphasized that by talking to others who had attended screening, they gained valuable information about the procedure, *Her sister in law, she went for breast screening, so she’s kind of explained to her, what happened, and how it’s done* (*Bushra via interpreter*). ‘*So, how does it make you feel, knowing that extra information before you are due to go?*’ (*Interviewer*). ‘*It’s helpful, awareness about our body changes and diseases, so it can help us also, when I know what it is, and what to expect*’ (*Bushra via interpreter*). Women expressed that breast screening and breast health should be regularly talked about amongst women in the community. Specifically, knowledge should be filtered through generations, enabling young women to become informed about the importance of screening, *‘ … knowledge needs to be passed on, should be talked regular, especially to your daughters, that this is very important, a vital part of your life…’ (Sadia via interpreter).* Women described using their experiences to encourage and support others when making their decisions to attend. However, not all women in their communities, especially older generations, were comfortable in discussing private appointments, potentially resulting in insufficient knowledge transfer.

#### Subtheme 2b: Ineffective screening materials

Only two women out of those who said they had attended screening recalled being able to access information about the NHSBSP when invited and understand what was going to happen at their appointment. The majority described having no prior knowledge, and attended their appointments naïve about what it would entail, *‘She didn’t have any knowledge about it, no, how it’s going to happen or anything, until she actually went there. It was kind of an eye-opener for her…’* (*Ushna via interpreter*). For these women, their limited literacy in English often resulted in valuable information being ignored, missed, or thrown away, rendering the information provided in this format as inaccessible and useless, *‘…because everything was in English I only read first few line and then I chuck it in the bin, the leaflet…’* (*Tahmina*). Instead, holding regular community events, in venues of familiarity such as GP surgeries, schools, and Muslim community centres, where women could come together to learn about breast screening was seen as a more effective way of disseminating crucial information, ‘*…invite them to an awareness day where you would disseminate the information at that venue […] As in the fact that well look, it’s just screening. And this is how it’s going to benefit you and this is the process then. And then that way they’ve had it orally*’ (*Fatima via interpreter*). Yet, women explained that leaflets are still valuable resources, but would be more likely to be read if they were distributed in a variety of languages.

#### Subtheme 2c: Incomprehensible terminology

The terminology used to describe breast screening including ‘screening’ and ‘mammogram’ are difficult to comprehend, *‘Cause breast screening, again, when that’s put together, when someone’s reading that […] it’s like an alien kind of a word, like screening what is it?’* (*Roshanah via interpreter*). It was explained that in the common languages spoken by the British-Pakistani community (i.e. Urdu), clear and direct translations of screening terminology is lacking. The uncertainty over what these terms denote caused some women to speculate about what would happen during their appointments, often resulting in inaccurate suppositions, *‘What is breast screening? Is it a screen that you go through, or is it like a room, is it like a shower room, what is it?’* (*Sadia via interpreter*). Women suggested that a glossary of screening terminology could be provided, or more common terminology (e.g. ‘X-ray’ or ‘scan’) could be used to reduce conjecture and facilitate understanding.

### Theme 3: Personal suppositions of breast screening

#### Subtheme 3a: Screening presents challenges to cultural beliefs

The necessity to reveal breasts to a stranger is a major deterrent to attending breast screening for British-Pakistani women, *‘ … she’s just saying, her religion, says the same, her culture, if you’re a daughter, you can’t be uncovered’* (*Ushna via interpreter*). It was apparent that if women knew that a female radiographer would be performing the mammogram, they would be more inclined to attend. Women made a distinction that, in their culture, for routine appointments such as screening, it is more appropriate to see a female healthcare professional; however, when events become more serious, the preservation of life becomes more important than cultural customs, ‘* … other ladies, they don’t like to be, like, be naked in front of other men, so that’s why they get a bit more shy. But, in our culture, like in Islam, if you’ve got a disease or something, or if you’ve got some serious problem, and if it’s a male doctor, then there’s no problem, you can go to him … *’ (*Iffat via interpreter*). In particular, one woman yet to be screened expressed that if a man were to perform the procedure she would not attend, *‘Well, if it’s a man, I prefer, I don’t go’* (*Namra*). To avoid this uncertainty, women explained that the presence of female *only* radiographers should be explicitly highlighted at events and in screening materials, to reassure women with specific cultural and religious beliefs.

#### Subtheme 3b: Screening is a symptomatic service

Despite receiving letters inviting women to screening, it was highlighted that many women view their attendance as unimportant, *‘So even though the letter might come through the door a lot of women disregard it and just feel like well, it's not really important. Do I really need to go? So they brush it to one side’* (*Fatima via interpreter*). Women suggested that this ambivalence towards screening may, in part, be due to the knowledge that women in their community hold about the *purpose* of breast screening. It was suggested that many British-Pakistani women view screening as a symptomatic service, rather than an early detection measure, with one woman explaining that she saw no merit in attending her appointment, ‘*First time she went, well, she was fit and healthy, and then her husband … she said to her husband, I don't really need to go [ … ] She didn't feel any kind of symptoms or anything like that, so there was no need for her to go*’ (*Zareen via interpreter*). This was evidenced further by another woman, who identified women in her community who only actively seek breast screening or healthcare when symptoms arise, *‘ … it’s really when they get the disease and they feel it’s [the breast] hard, then they will go, before that they think they don’t need to go’* (*Sara via interpreter*).

## Discussion

By exploring British-Pakistani women’s experiences and views of the NHSBSP, this study has uncovered a number of modifiable barriers that could be addressed at a service level to improve the programme for underserved populations. Specifically, language barriers were highlighted as particularly problematic, with information being missed, as well as autonomy and privacy being compromised. Women also highlighted that communication about the service could be improved, by focusing on alternative dissemination strategies, for example, providing information via community networks. Additionally, specific attention should be aimed toward making the terminology used to describe breast screening more accessible, as well as providing explicit publicity for available translation and interpretation services. Women also pointed out the challenge that screening presents to modesty, with women reluctant to attend, unless the NHS personnel are female. Incorrect knowledge regarding the purpose of screening was also described as a major contributor to low attendance.

A strength of this study is it enabled women from a particular underserved population with low uptake to breast screening to express the specific personal and service level issues they encounter. Nevertheless, with ethnicity and language spoken not routinely collected by the NHS^21,22,23^, there was no systematic way to reliably identify disengaged women. Furthermore, minority ethnic groups are often described as ‘hard to reach’ in research.^32^ In this study 19 British-Pakistani women were interviewed. Although we believe that data sufficiency was achieved, interviews with women who disengage with screening could have yielded more insight, as well as specifically recruiting women who are unable to read and write. The researchers who conducted the interviews were not of the same cultural background as the women, and interpreters were therefore employed. However, an advantage of the researchers’ naivety to the participants’ cultural traditions and beliefs meant that they engaged in more in-depth enquiry, with layers of meanings explored to facilitate understanding, rather than drawing assumptions based on shared cultural knowledge.

A specific challenge of communicating with women via an interpreter was that the researchers could not guarantee that verbatim translations were being obtained. To mitigate against this, interpreters were thoroughly briefed about the aims of the study prior to commencing the interview. The need for accuracy when translating for and to the women was also stressed. If time and resources had allowed, an independent interpreter could have been employed to listen to the recordings of the interviews, to assess the accuracy of the translations. Nevertheless, a major strength of employing interpreters was their shared cultural background with the participants. For example, on arrival at participants’ homes, traditional forms of welcome were exchanged. Therefore, via the interpreter, the researchers were able to build rapport, and attain cultural sensitivity.

A recurring issue underpinning these interviews was the incongruence between languages and the challenge women face in communicating with healthcare professionals, maintaining privacy, and understanding the importance of screening materials and health messages. Central to the NHS constitution is the right to privacy, confidentiality, and the provision of information to enable informed choice.^33^ To facilitate this, translation and interpretation services are widely available across NHS trusts, but this study has shown a lack of awareness of these services. Access to these services need to be more widely advertised, especially via screening invitations, to enable women to receive unfiltered health messages and to attend appointments unaccompanied.

Our results show that information about breast screening provided in English is largely ignored, resulting in misunderstandings and speculations about the screening procedure. Via NHS England’s Accessible Information and Communication Policy, information materials are made available online in different languages.^34^ However, with ‘language spoken’ not being formally collected by NHS services,^21–23^ there is currently no pro-active way to distribute tailored and translated materials to service users. This policy therefore only partly addresses the problems of inequalities and facilitating informed choice amongst underserved communities. The health service and screening services should begin to collect data on service users’ preferred communication, to promote understanding, preserve autonomy and privacy, and enable informed choice.^22^ Asking patients at the point of GP registration to specify their preferred method of communication and language may enable this change to be implemented. For example, for those who have limited or no literacy skills in English or their spoken language, a telephone call, via an interpreter, may be the most effective form of communication when being invited for appointments such as breast screening.

This study highlighted the problem that the terminology used to describe screening is not directly translatable or understandable. This reinforces research which suggests that translations are not always reliable.^18^ Furthermore, women explained that screening in their community is often seen and described as a symptomatic service, rather than a service for the prevention and early detection of breast cancer. The incomprehensible and inaccessible terminology used to describe screening could partly account for this misunderstanding. Small changes to screening materials could be made to facilitate understanding, and thereby to reduce conjecture. Direct involvement with women from underserved populations in the creation of screening materials would produce more accurate linguistic translations, providing consistent and accessible health messages across languages.

The value of community dialogue was clear in this population of women. It was suggested that community-based education events would provide a comfortable and safe environment for women to come together to learn about breast screening. Here, procedural information and the purpose of screening could be disseminated to facilitate understanding, especially amongst those who are unable to access screening materials (i.e. those illiterate in English or their spoken language). Indeed it has been suggested in this study and previous literature, that holding educational events in locations of familiarity would be advantagious.^18^ In this study local schools were suggested as potential locations, as women do not have to make unscheduled trips to attend. To facilitate communication between healthcare professionals and women at these events, multilingual lay health workers should be used in an attempt to improve knowledge and screening uptake.^35^

The necessity to show intimate body parts has been recognized as a difficult obstacle to overcome, as modesty and respect are highly valued in this community.^5^,^15^,^16^,^18^ There is an opportunity for screening materials to address cultural sensitivities, and to reassure women whose values are incongruent with Western medical practices.^15^ In the UK, radiographers performing mammograms are female, but this was not common knowledge. There is further opportunity to address this, by reassuring women that breast screening is a female only environment. Where cervical screening may be performed by a male healthcare professional, South Asian women have expressed their discomfort.^36^,^37^ Without explicit information, we argue that women are given no reason to believe that breast screening will not be performed by a male radiographer. Information which directly communicates breast screening as a female only environment would probably reduce this concern, not only for British-Pakistani women, but for all women wishing to access the service.

## Conclusions

This research has shown that inequity in access continues to be challenging for British-Pakistani women. To mitigate the challenges presented by language difficulties, the breast screening service should provide explicit information signposting women to the NHS Translation and Interpretation Service, and should, in addition, involve women in the creation of screening materials. Positive changes to engagement could be made by utilizing family and community networks, via health awareness events hosted in facilities across the community. At these events, specific attention should be given to explaining the screening environment and purpose of screening. The NHSBSP and, more broadly, the health service should begin to routinely record the service user’s preferred form of communication, to enable underserved populations to receive tailored health materials. Should this be implemented, further research should aim to identify non-attenders to breast screening across different underserved populations, to gain different perspectives on disengagement. Doing so would add nuanced insight into screening disengagement and contribute positively to the fundamental changes needed to make breast screening accessible to underserved populations.

## Authors’ note

The dataset analysed is not publicly available, as the data may contain information that would compromise participant consent. Contact the corresponding author for more information.
